# Anti-CS1 × Anti-CD3 Bispecific Antibody (BiAb)-Armed Anti-CD3 Activated T Cells (CS1-BATs) Kill CS1^+^ Myeloma Cells and Release Type-1 Cytokines

**DOI:** 10.3389/fonc.2020.00544

**Published:** 2020-05-05

**Authors:** Lawrence G. Lum, Archana Thakur, Abdalla Elhakiem, Lena Alameer, Emily Dinning, Manley Huang

**Affiliations:** Division of Hematology and Oncology, Bone Marrow Transplantation and Department of Medicine, University of Virginia Cancer Center, Charlottesville, VA, United States

**Keywords:** bispecific antibody, elotuzumab, activated T cells, OKT3, multiple myeloma, cytotoxicity, cytokines, chemokines

## Abstract

**Background:** Multiple myeloma (MM) remains incurable despite *significant* advances in chemotherapy, targeted therapies, and immunotherapy. Bispecific antibody (BiAb)-armed activated T cells (BATs) have been developed for targeting and treatment of solid and hematologic malignancies. BATs are serial killers of tumor cells, secrete Th_1_ cytokines, and induce adaptive cellular and humoral immune responses in patients (pts). This study provides preclinical data using bispecific anti-CS1 (elotuzumab) × anti-CD3 (OKT3) antibody (CS1Bi)-armed activated T cells (CS1- BATs) that provide a strong rationale for applying CS1-BATs to pts with MM.

**Methods:** CS1-BATs and unarmed activated T cells (ATC) were incubated with MM cell targets at various effector to target ratios (E:T) in a quantitative flow cytometry-based assay to determine the degree of cell loss relative to target cells incubated without ATC. ATC from up to 8 normal donors were armed with various concentrations of CS1 BiAb and tested against 5 myeloma cells lines for CS1-BATs-mediated killing and release of Th_1_ cytokines, chemokines and granzyme B.

**Results:** CS1-BATs from normal donors killed each of 5 MM cell lines proportional to E:T ratios ranging between 1:1 and 10:1 and arming concentrations of 12.5 to 50 ng/million ATC, which was accompanied by release of Th_1_ cytokines, chemokines and granzyme B. CS1-BATs prepared from MM pts' peripheral blood mononuclear cells (PBMC) showed increasing cytotoxicity and T cell expansion over time against ARH77 MM cells. The optimal arming dose of CS1Bi is 50 ng/10^6^ ATC.

**Conclusions:** These data demonstrate the therapeutic potential of CS1-BATs-mediated cytotoxicity and Th_1_ cytokines release at low E:T and support advancing their clinical development in pts with MM.

## Introduction

Multiple myeloma (MM) is the second most common hematologic malignancy. Patients are the most sensitive and responsive to the first line of therapy, which provides the highest chance of achieving minimal residual disease (MRD) negativity. With subsequent lines of therapy, the depth and duration of response typically lessens and many patients ultimately become refractory to treatment. With the introduction of proteasome inhibitors (PIs), immunomodulatory agents (IMiDs), histone deacetylase (HDAC) inhibitors and monoclonal antibodies (mAb), the number of patients achieving 5 year survival in 2019 is now over 50% (seer.cancer.gov). Despite the effectiveness of combination therapies, autologous stem cell transplant (autoSCT) and maintenance, MM remains an incurable disease. Non-toxic specific anti-MM approaches that induce long-term anti-MM immunity are needed to purge residual CD34-CD138- clonogenic cells from the marrow to improve progression-free survival (PFS) and overall survival (OS). The goal of therapy is to achieve the deepest possible response with MRD negativity since the probability of long-term remission is highest in MRD negative patients (1). Signaling lymphocytic activation molecule family 7 (SLAMF7) is a cell surface receptor, also called cell-surface glycoprotein CD2 subset 1 (CS1), expressed at high levels on MM cells and at lower levels on NK cells where it acts as an activating receptor. The overexpression of CS1 in MM in more than 90% of cases, irrespective of cytogenetic abnormalities (2), makes it an attractive target for immunotherapy. Elotuzumab (Elo) is a humanized immunoglobulin G1 immunostimulatory antibody targeted against CS1. It works by activating natural killer cells, mediating antibody-dependent cell-mediated cytotoxicity (ADCC), and may further enhance cytotoxicity by promoting CS1-CS1 interactions between NK cells and CS1+ target cells independent of ADCC (3). Interestingly, Elo does not directly mediate anti-MM activity as a single agent, but works synergistically with IMiDs (4).

Our strategy combines the cellular cytotoxicity of ATC with the anti-CS1 targeting specificity of Elo. OKT3, which is directed at the activating CD3-epsilon chain of the T cell receptor (TCR), is chemically heteroconjugated to anti-CS1 to form CS1Bi. Arming of *ex vivo* expanded ATC with CS1Bi converts each ATC into an anti-CS1 cytotoxic T lymphocyte (CTL). Although we have reported preclinical work, as well as clinical trials, that arm ATC with (a) anti-CD3 x anti-HER2 BiAb (HER2 BATs) for the treatment of breast and prostate cancer (5, 6), and (b) anti-CD3 x anti-CD20 BiAb (CD20 BATs) for the treatment non-Hodgkin's lymphoma (7) and MM in combination with stem cell transplantation, specific targeting to MM lines by CS1-BATs has not been shown. Armed ATC derived from normal donors not only kill repeatedly, but secrete Th1 cytokines, chemokines (8) and granzyme B when a BiAb bridge synapse is formed between the effector ATC and its target.

## Methods

### Approach

The strategy for producing heteroconjugated BiAb for arming ATC involves crosslinking OKT3 with a 10-fold molar excess of Traut's reagent and anti-CS1 (elotuzumab) with a 4-fold molar excess of Sulpho-SMCC according to manufacturer's instructions (9) (step 1), mixing the two cross-linked antibodies overnight at 4°C to produce heteroconjugated CS1Bi (step 2), arming the *ex vivo* expanded ATC with CS1Bi (step 3), and co-culturing the CS1-BATs with MM cell line targets leading to cytotoxicity and cytokine release (step 4).

### Activated T Cells

PBMC from normal subjects were obtained with informed and written consent under University of Virginia (UVA) Institutional Review Board (IRB)#18904. PBMC from MM pts were obtained with informed and written consent under UVA Orien IRB HSR 18445 and Wayne State University (WSU) IRB-approved protocol 2008-106 (NCT00938626) (10). PBMC were isolated by Ficoll-Hypaque (Lymphocyte Separation Medium from Corning) and stimulated with OKT3 at 20 ng/ml and expanded in RPMI-1640 containing 10% fetal calf serum and IL-2 (100 IU/ml) as described (8). Unseparated ATCs were armed between 10 and 15 days of culture, most often between 12 and 14 days. Historically, patients' ATC cultures consisted primarily of CD3+ cells, with a small percentage of CD56+ cells. In the phase 1 breast cancer trial, the average composition of 17 patients' ATC products for CD3, CD4, and CD8 cells were 86.7% (+/– 13.5), 52.4% (+/– 15.2), and 34.6% (+/– 15), respectively (5); and for 12 myeloma patients were 94.6% (84.4–98.3), 66.2% (24.8–81.1), and 39.1% (10.2–71.3), respectively (with a mean CD3–/CD56+ of 11.6%, ranging from 0.35 to 63.7) (10).

### Multiple Myeloma Cell Lines and Monoclonal Antibodies

The MM cell lines RPMI8226, ARH77, L363, and MM.1S were purchased from ATCC, Manassas, VA. OPM2 was purchased from DSMZ, Germany. OKT3 is an anti-CD3 immunoglobulin G2a (IgG2a) (Miltenyi Biotech). Elo was obtained commercially. OKT3 was chemically heteroconjugated with Elo as described (9).

### Quantitative Flow Cytometry-Based Specific Cytotoxicity Assay

**Figure 1:**
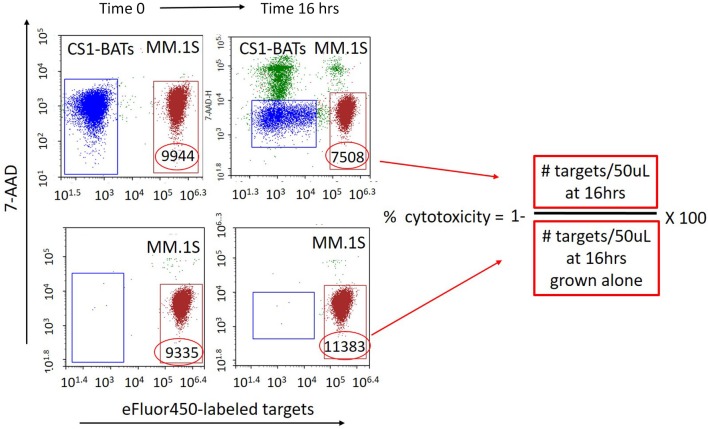
Quantitative flow cytometry-based assay for measuring specific cytotoxicity against multiple myeloma and other non-adherent cell targets. eFlour450-labeled cell targets are cultured alone or in the presence of either BATs or unarmed ATC for 16 h (or longer). The absolute number of target and effector cells in a fixed volume (50 μL) of the test culture is measured upon initial mixing and at a later time point(s) from the same culture. The number of target cells cultured alone is used as a reference for calculating cytotoxicity as a result of co-culturing with ATC. The number of surviving target cells (eFluor450 positive/7-AAD negative) per fixed volume is used to calculate the percent cytotoxicity as [1− (# live targets in ATC co-cultures divided by the number of live targets in parallel cultures grown without ATC)] × 100. Numbers within the gates represent the # of live target cells/50 μL at each time point. *Top left:* BATs plus eFluor450-labeled targets at Time 0. *Bottom left:* eFluor450-labeled targets alone at Time 0. *Top right:* BATs + eFluor450-labeled targets at Time 16 h. *Bottom right:* eFluor450-labeled targets alone at Time 16 h. In this example, cytotoxicity of CS1-BATs against MM.1S myeloma cells is [1-(7508)/11383)] × 100% = 34% at 1:1 E:T.

First attempts to measure the cytotoxicity of CS1-BATs using standard 4 h ^51^Cr-release assays showed minimal activity against MM cells even at 25 E:T. Therefore, a more sensitive quantitative assay was developed using flow cytometry in which the concentration of both effector T cells and target cells was measured in fixed volume aliquots (50 μL) before and after 16 h (or more) of culture using an ACEA Biosciences NovoCyte flow cytometer. Target cells are fluorescently labeled with eFfluor 450 (Invitrogen) according to manufacturer's instructions, resuspended at 0.8 × 10^6^ cells per mL, and added to 24 well culture plates in 300 μL of media. T cells are resuspended to provide the designated E:T ratios based on the addition of 300 μL to the target cells. After thoroughly mixing the cells, 120 μL is placed into a counting tube, 7-ADD added, and the cells acquired on the cytometer to establish the baseline E:T ratio. Cells are first gated by forward and side scatter to capture the T cell and myeloma cell line populations. At the final time point, the co-cultured cells are again thoroughly mixed by gentle pipetting prior to sampling. Figure 1 shows a representative example of the gating used to calculate the specific cytotoxicity directed at MM.1S cell line using 7-AAD (live/dead staining) and eFlour 450-labeled targets. The formula for analysis is as follows: Number of cells/gate are the number of cells per 50 μL of the test culture volume assessed at baseline and at subsequent time points. % cytotoxicity = 1− [# targets incubated with effector T cells at a given time point/# targets at the same time point cultured without effectors] × 100%. Due to the ability to finely measure the E:T ratios in each well, the closest integer value for a donor set is presented in the figures, with the actual range of E:T indicated in the figure legends. For multiday studies, replicate wells are prepared so that the experimental and target-only wells are collected only once at the designated time points.

### Quantitation of Cytokines/Chemokines

CS1-BATs or unarmed ATC were co-cultured in the presence of MM cell lines targets, and cytokines, chemokines and granzyme B in the cell-free supernatants were quantitated using the Luminex system. The values are reported in pg/ml (ng/mL for granzyme B) of cell supernatants.

### Statistical Analyses

All values are expressed as means ± SD. Mean values were compared using Student's *t*-tests (Prism software) with *p* < 0.05 considered significant for parametric paired samples.

## Results

### Production of Chemically Heteroconjugated Anti-CD3 × Anti-CS1 (CS1Bi)

**Figure 2:**
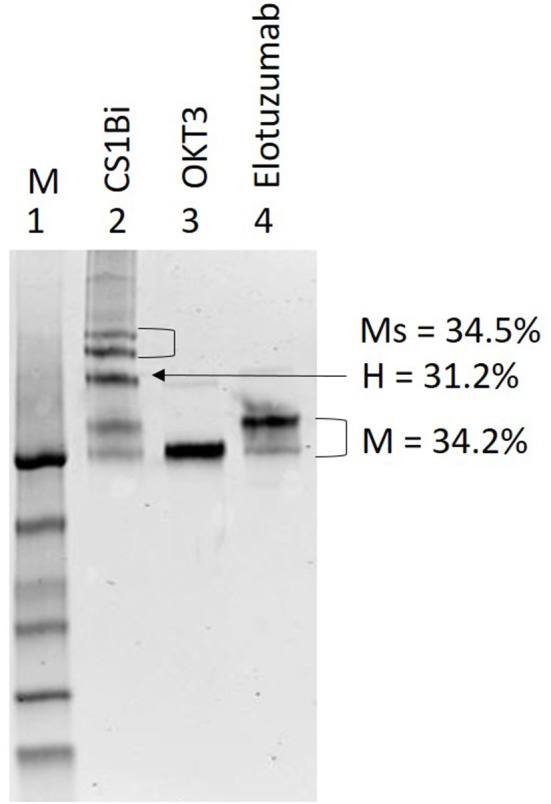
SDS-PAGE of CS1Bi heteroconjugation product. *Lane 1*: molecular weight markers; *Lane 2*: CS1Bi product containing unconjugated monomers, heterodimer, and multimers forms; *Lane 3*: OKT3; *Lane 4:* elotuzumab. M, monomers; H, heterodimers; Ms, multimers.

The Coomassie stained non-reducing gel in Figure 2 shows the results of the heteroconjugation. Lane 2 of the gel shows the CS1Bi product adjacent to unconjugated OKT3 (lane 3) and Elo (lane 4). Scanning densitometry of the gel shows 31.2% dimer, 34.2% monomers, and 34.5% multimers.

### Antibody and BiAb Binding to MM Cell Lines and ATC

**Figure 3:**
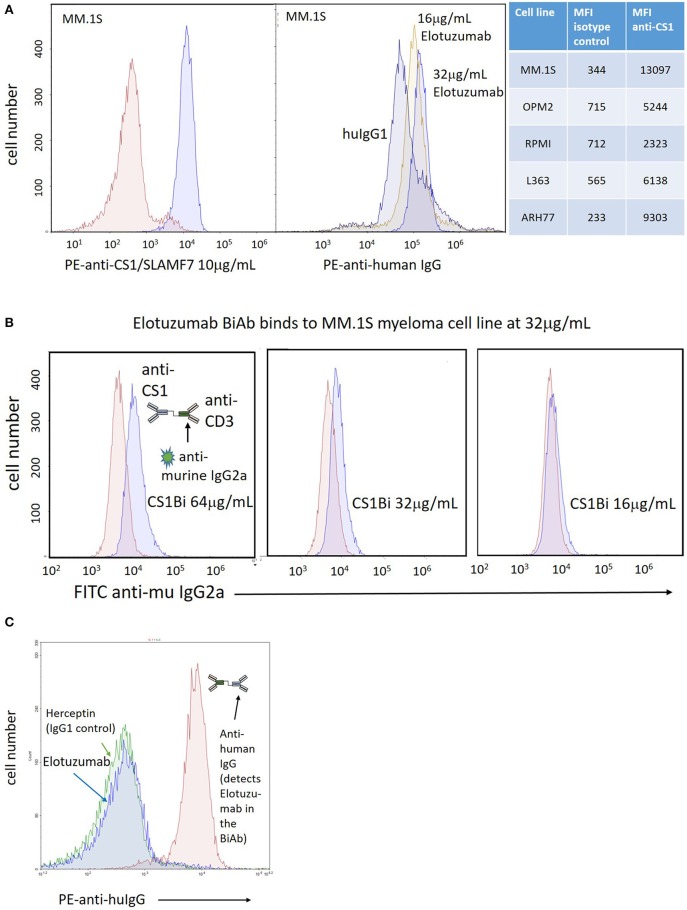
Expression of CS-1 on MM cell lines. **(A)** *Left panel*: Right peak shows binding of PE-anti-CS1 (Abcam, 95827; clone 162) at 10 μg/mL and 0.4 million MM.1S cells after incubation in 100 μL phosphate buffered saline (PBS)/0.2% bovine serum albumin for 20' at 4°C; Left peak shows binding of PE-isotype control. *Right panel*: Right peaks show relative binding of Elo to MM.1S cells incubated at 16 μg/mL and 32 μg/mL relative to 32 μg/mL human IgG1 (left peak), stained with PE-anti-human IgG (Biolegend, 409304; clone HP6017). The relative binding of the Abcam PE-anti-CS1 vs. isotype control for 5 MM cell lines is shown in the table to the right of the histogram panels. **(B)** Arming titration of CS1-BATs against MM.1S MM cells. Each panel shows binding of CS1Bi by FITC anti-murine IgG2a at either 16, 32, or 64 μg/mL CS1Bi/0.4 million ATC after incubation in 100 μL phosphate buffered saline (PBS)/0.2% bovine serum albumin for 20' at 4°C. The histograms are overlaid against staining of MM.1S by 8, 16, or 32 μg/mL OKT3, respectively, which represents the relative amount of OKT3 in the CS1Bi product. **(C)** Binding of CS1Bi to ATC. CS1Bi was incubated at 500 ng/mL/0.4 million ATC in 100 μL for 20' at 4°C followed by staining with PE-anti-human IgG. Right peak shows the binding to Elo in the bound BiAb. The left peaks show background binding of ATC incubated with Elo or Herceptin (both human IgG1 isotype) at 2 μg/mL/0.4 million ATC.

To confirm CS1 expression on MM cells, the MM cell lines were stained with a commercial PE-conjugated anti-CS1 mAb, or Elo followed by secondary staining with PE anti-human IgG. There is clear CS1 expression on the MM.1S myeloma cell line at a concentration of 10 μg/mL of the PE-conjugated mAb (Figure 3A, left panel); binding of Elo was detected at 16 and 32 μg/mL (Figure 3A, right panel). The mean fluorescent intensity (MFI) for anti-CS1 binding for the 5 cell lines is tabulated in Figure 3A. Binding of the CS1Bi to MM.1S myeloma cells was detected using a FITC-conjugated anti-murine IgG2a antibody to detect OKT3 in the attached BiAb; OKT3 is barely detectable above the isotype control at 32 μg/ml CS1Bi and is clearly detectable at 64ug/mL (Figure 3B). In order to determine the ability of CS1Bi to bind to ATC, ATC were armed with CS1Bi at 500 ng/mL, and also incubated with Elo (2 μg/ml) and anti-HER2 antibody (Herceptin^®^, human IgG1, 2 μg/ml) as negative controls for human IgG1 binding. Flow cytometry confirms that the CS1 portion of the BiAb can be strongly detected on ATC using PE-anti-human IgG (Figure 3C).

### Dose Titrations to Determine Optimal Arming Concentration of CS1Bi

**Figure 4:**
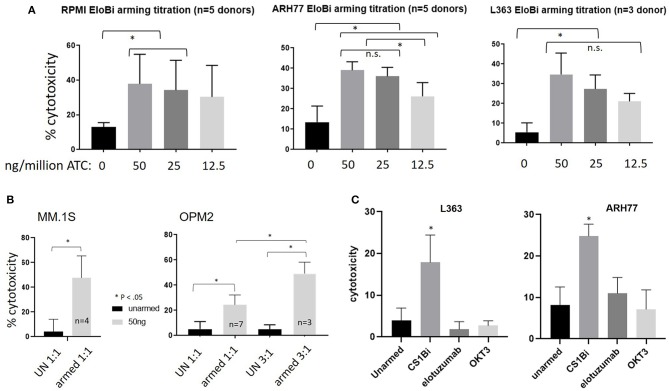
**(A)** Cytotoxicity of differentially armed BATs. Normal donor ATC were unarmed (UN) or armed with 12.5, 25, or 50 ng/10^6^ ATC and tested against RPMI (*Left panel; 1 to 1.5 E:T, n* = *5*), ARH77 (*Middle panel; 1.5 to 2.1 E:T, n* = *5*) and L363 (*Right panel; 1 to 1.5 E:T, n* = *3*) cells in a 16 h cytotoxicity assay. The difference between armed and unarmed ATC (UN) was significant (*p* < 0.05) for 50 and 25 ng/10^6^ ATC for all three cell lines, as well as for 12.5 ng/10^6^ ATC for ARH77 and L363 cells. ^*^P < 0.05. Arming at 50 and 25 ng/10^6^ ATC was significantly different than 12.5 ng/10^6^ for ARH77 cells. **(B)** Cytotoxicity of CS1-BATs against MM.1S and OPM2 MM cells. Unarmed (UN) BATs or BATs armed with 50 ng/10^6^ ATC were incubated for 16 h with MM.1S cells (*Left panel; 0.8 to 1.1 E:T, n* = *4)* and with OPM2 cells (*Right panel; 0.7 to 1.36 E:T, n* = *7; and 3.1 to 3.6 E:T, n* = *3*). MM.1S were highly sensitive to CS1-BATs at 1:1 E:T. OPM2 cells were relatively resistant at 1:1 E:T, with increased cytotoxicity at 3:1 E:T. (^*^P < 0.05). **(C)** ATC were armed with CS1Bi (50 ng/10^6^ ATC), elotuzumab (25 ng/10^6^ ATC), or OKT3 (25 ng/10^6^ ATC) and tested for cytotoxicity against L363 and ARH77 cells at 1:1 to 2:1 E:T in a 16 h flow-based assay. CS1-BATs showed significantly greater killing (^*^P < 0.05) than either unarmed, elotuzumab-armed, or OKT3-armed ATC.

In order to establish the optimum arming concentration of CS1Bi, the ATC were left unarmed or armed at 12.5, 25, and 50 ng of CS1Bi/10^6^ ATC and tested for cytotoxicity directed at RPMI8226, ARH77, and L363 MM cell lines at a 1 to 1.5:1 E:T (Figure 4A). Cytotoxicity directed at RPMI 6226 (Figure 4A, left panel) peaked just below 40%. In the ARH77 experiment, the cytotoxicity appeared to plateau around 40% at ≥25 ng of CS1Bi/10^6^ ATC arming dose (Figure 4A, middle panel). There were no significant differences between the amount of cytotoxicity mediated by ATC armed with 12.5, 25, and 50 ng/10^6^ ATC in RPMI6226; however the 50 and 25 ng/million ATC conditions were significantly different than unarmed ATC. The experiments in the L363 cell line showed that the dose titration continued to increase to ~35% at an arming dose of 50 ng/10^6^ CS1Bi and that all arming doses were significantly greater than for unarmed ATC (Figure 4A, right panel).

CS1-BATs armed with 50 ng CS1Bi/10^6^ ATC were also very effective against MM.1S cells (Figure 4B, left panel), while OPM-2 cells were the least sensitive line tested to CS1-BATs at 1:1 E:T, with increased killing at 3:1 E:T (Figure 4B, right panel). Although CS1-BATs showed increased cytotoxicity (>90%) at higher E:T, we focused on lower E:T to better distinguish the effects of the arming titration. Thus, the amount of specific cytotoxicity was significantly increased over unarmed ATC in all 5 cells lines (*p* < 0.05). Based on these results, and similar to our other BATs products, the clinical arming dose will be 50 ng of CS1Bi/10^6^ ATC.

The ability of elotuzumab and OKT3 alone to redirect the cytotoxicity of ATC was tested by “arming” ATC with each antibody at the same concentration present in the CS1Bi preparation (25 ng per million ATC). Figure 4C shows that neither antibody was able to significantly increase the degree of cytotoxicity above that of unarmed ATC against L363 and ARH77 cells.

### Cytotoxicity of CS1Bi-Armed PBMC

**Figure 5:**
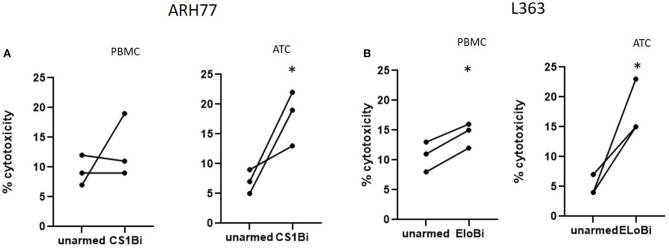
Cytotoxicity of CS1Bi-armed PBMC from normal donors. PBMC and ATC from 3 normal donors were armed with 50 ng/million cells and co-cultured with ARH77 and L363 cells for 16 h at 2:1 E:T. Each donor is represented by a pair of connected dots showing the results of unarmed vs. armed cells. **(A)** CS1Bi-armed PBMC vs. ATC for ARH77 cells. **(B)** CS1Bi-armed PBMC vs. ATC for L363 cells (^*^P < 0.05).

The relative ability of unactivated PBMC to mediate anti-MM cytotoxicity was tested by arming total PBMC at 50 ng/million cells and compared to that of CS1Bi-armed ATC (Figure 5). Either BiAb-armed or unarmed cells from 3 donors were added to ARH77 or L363 cells. The percentages of lymphocytes in these 3 PBMC samples were 60, 78, and 82. Against ARH77 cells, the cytotoxicity of the armed vs. unarmed PBMC was unchanged for 2 out of 3 donors, with the third showing an increase in cytotoxicity. This result is similar to what was reported with PBMC armed with anti-Her2 x anti-CD3 BiAb (11). Against L363 cells, 3 out of 3 donors showed significantly higher cytotoxicity when armed with CS1Bi.

### Cytotoxicity of CS1 BATs Is Not Blocked by Free Elotuzumab

**Figure 6:**
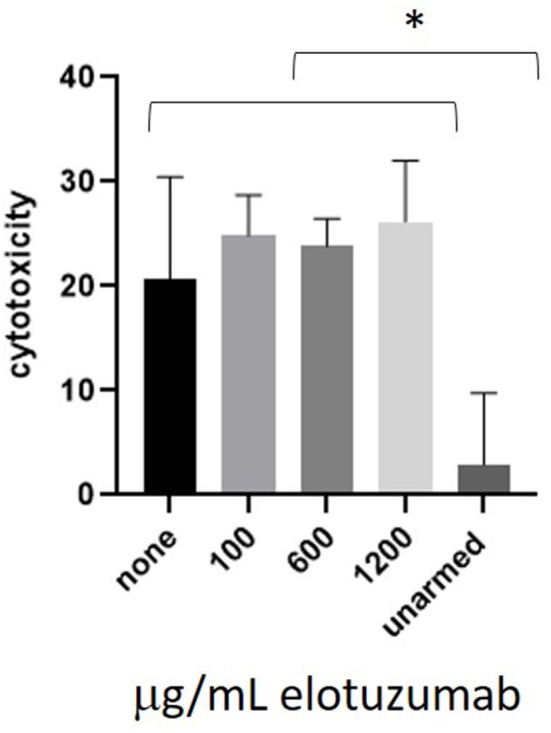
Co-culture of CS1 BATs with L363 cells in the presence of elotuzumab. ATC from 3 normal donors were armed with 50 ng CS1Bi and co-cultured with L363 at an E:T of 1:1 (^*^P < 0.05).

CS1 BATs were co-cultured with L363 cells in the presence of 100, 600, or 1,200 mcg/mL elotuzumab, which represent concentrations below, at, and above the maximum blood concentration in pts (Figure 6). There was no difference between the untreated and elotuzumab-treated groups and all CS1 BATs concentrations were significantly greater than for unarmed cells.

### Induction of Th_1_ Cytokine and Chemokine Release Upon Binding Target Cells

**Figure 7:**
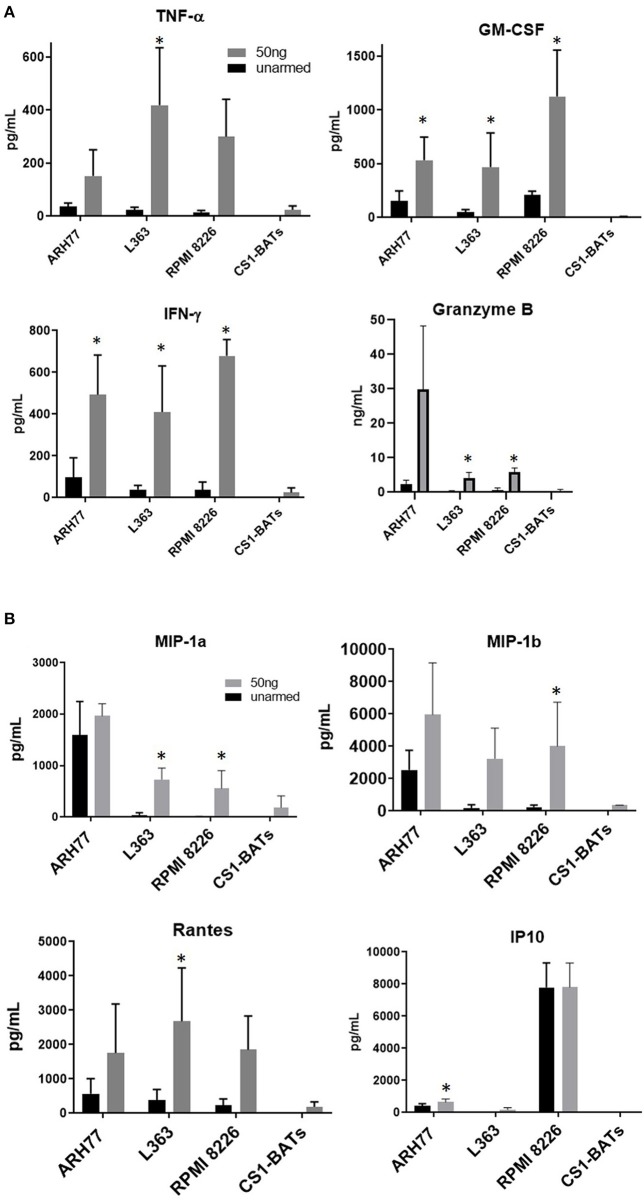
Cytokine production upon engagement of CS1- BATs. Cell-free supernatants from 16 h co-cultures of multiple myeloma cell lines with unarmed ATC, CS1 BATs armed with 50 ng/10^6^ ATC at 1:1 E:T (*RPMI, 1.1 to 1.5 E:T; L363, 1.1 to 1.5 E:T)* or 2:1 E:T (*ARH77, 1.1 to 1.8 E:T)* (*n* = 4 donors), or CS1-BATs cultured alone (*n* = 3 donors) were analyzed for production of cytokines, granzyme B, and chemokines. **(A)** Average levels of IFN-γ, TNF-α, GM-CSF (pg/ml), and granzyme B (ng/mL) (*n* = 4 donors) are summarized in the 4 panels. **(B)** Chemokine production by CS1-BATs. The levels of type-1 chemokines (MIP1-a, MIP1-b, Rantes, and IP10) present in the same supernatants as in **(A)** are shown for each of the cell lines tested (pg/mL). ^*^P < 0.05.

Overnight co-cultures of unarmed ATC and ATC armed with 50 ng of CS1Bi/10^6^ ATC, were performed to assess the induction of Th_1_ cytokines, chemokines and granzyme B secretion upon CS1-BATs engagement with RPMI 8226, ARH77, and L363 cells. The amounts of IFN-γ, TNF-α, GM-CSF, and granzyme B secreted during a 16 h co-culture increased as a function of CS1Bi arming dose by CS1-BATs produced from 4 normal subjects. (Figure 7A). The 50 ng/10^6^ ATC arming dose consistently induced more secretion of the respective cytokines and granzyme B vs. unarmed ATC. Significantly elevated levels of chemokines were seen against some of the cell lines for of MIP1-α, MIP1-β, RANTES, and IP10 (Figure 7B). CS1-BATs cultured alone did not produce significant amounts of any of the factors tested. ^*^P < 0.05.

### Specific Cytotoxicity Mediated by ATC of MM Patients

**Figure 8:**
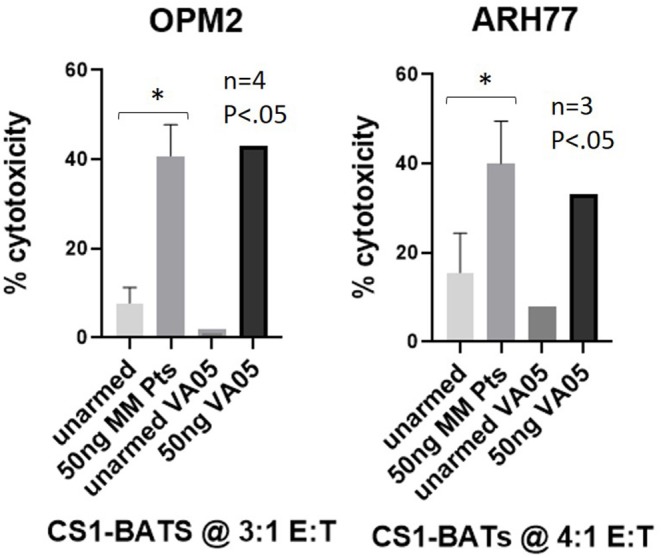
Cytotoxicity of MM pt-derived CS1-BATs. Cryopreserved PBMC from 2 MM pts from WSU and 2 pts from UVA were used to prepare ATC by activation with OKT3 and expansion in IL2-supplemented media for up to 14 days. ATC were armed with CS1Bi at 50 ng/10^6^ ATC and co-cultured with OPM2 (*n* = 4) at 2.8–3.6 E:T and ARH77 (*n* = 3) cells at 3.5–4.0 E:T for 16 h. CS1-BATs from a normal donor VA05 (3.2–3.8 E:T) were included as a positive control for each cell line. The level of cytotoxicity of the pt samples was comparable to that of the normal donor performed in parallel (^*^P < 0.05).

To test whether CS1Bi could trigger ATC produced from cryopreserved PBMC from 4 MM Pts, CS1-BATs armed with 50 ng of CS1Bi/10^6^ ATC were tested for cytotoxicity directed at OPM2 and ARH77 MM cell lines at 3:1 and 4:1 E:T, respectively. Significant cytotoxicity was observed against both cell lines, which was comparable to a normal donor (VA05) tested in parallel (Figure 8).

### Sequential Cytotoxicity by CS1-BATs

**Figure 9:**
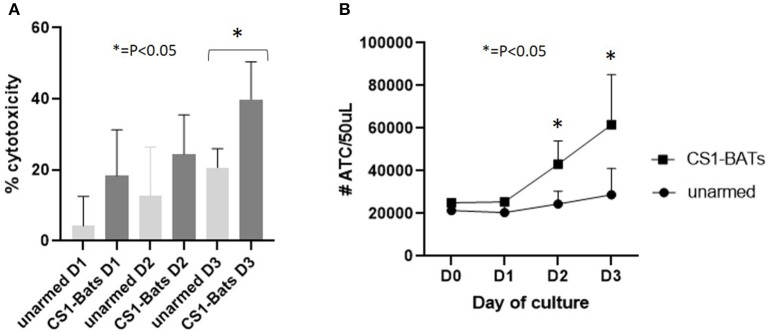
Continuous cell killing and effector cell expansion of MM pt CS1-BATs. CS1-BATs from 3 MM pts were co-cultured with ARH77 target cells at 1:1 E:T (1.1 to 1.5) for 3 days. Duplicate samples for BATs, unarmed ATC, and ARH77 cells grown alone were analyzed for each day of culture to determine relative cytotoxicity and number of ATC for each day of culture. **(A)** Cytotoxicity increased on each successive day, and was statistically different for CS1-BATs vs. unarmed ATC on day 3. **(B)** A similar trend was observed for the total number of ATC present, with the number in CS1-BATs-treated wells significantly higher at days 2 and 3 (^*^P < 0.05).

In order to show that CS1-BATs are capable of killing multiple times and divide in response to engaging MM cells, CS1-BATs from 3 MM pts were incubated at 1:1 E:T with ARH77 cells supplemented with 100 u IL2/million ATC in the original culture media, and assayed over 3 days to determine the % cytotoxicity and relative number of ATC over time. The % cytotoxicity doubled after 3 days (Figure 9A; *p* < 0.05), accompanied by a 2.46-fold increase in the starting concentration of ATC relative to unarmed ATC (Figure 9B; *p* < 0.05 for days 2 and 3). The relatively lower cytotoxicity of these samples vs. normal donors at 1:1 E:T (Figure 4A) is not unexpected given they were derived from MM patients as well as that 2 out of the 3 PBMC samples had been frozen for 8-9 years. This result shows that upon activation, CS1-BATs are stimulated to divide and are capable of continuous killing. Therefore, even at lower E:T, CS1-BATs can provide an extended effect against MM cells that can be further enhanced through multiple infusions of BATs to promote a cytotoxic anti-tumor microenvironment over time as was seen with HER2-BATs-treated breast cancer pts (5).

## Discussion

This study shows that arming ATC with 25–50 ng of CS1Bi/10^6^ ATC can generate highly effective cytotoxic T cells directed at CS1 on MM cell lines. *Ex vivo* arming ATC avoids the need to administer large quantities (mg/kg) of BiAb and, more importantly, would likely avoid cytokine release syndrome (CRS) associated with the infusion of anti-CD3 targeting BiAbs. This strategy utilizes humoral antibody targeting to mediate non-MHC restricted cytotoxicity by ATC. Secretion of Th_1_ cytokines upon binding of the effector cells to the myeloma cells not only augments tumoricidal activity directed at the malignant B cells, but may increase local cytokine and chemokine secretion that leads to shifting the tumor microenvironment to recruit endogenous immune effectors and induce an endogenous immune response.

Although the chemical heteroconjugation does not produce pure dimers of OKT3 × Elo, the preparation contains enough dimers and multimers to arm ATC converting each ATC into a CS1-targeted CTL. The titration studies determined the “effective” dose of CS1Bi to be 50 ng/10^6^ cells. The concentration of anti-CS1 antibody needed to demonstrate the presence of CS1 on the MM cells suggests that CS1Bi is highly effective at triggering BATs against very low amounts of CS1 antigen on the target cells. Flow cytometry data confirmed that the CS1Bi could be easily detected on the ATC. The CS1-BATs were cytotoxic to all 5 MM cell lines even though CS1 is not highly expressed. These observations parallel our earlier study that showed that HER2Bi at an arming dose of 50 to as little 5 ng of HER2Bi/10^6^ ATC was not only capable of binding but also mediating specific cytotoxicity and release of Th1 cytokines (IFN-γ, TNFα, and GM-CSF) when the HER2 BATs engaged the HER2 negative cell line MCF-7 (9). An analysis of CS1 expression on MM pts and cell lines showed that most of the cell lines tested expressed less CS1 than pts (12). However, our experience with solid tumor lines does not show a direct correlation between antibody target expression and overall cytotoxicity or release of cytokines, e.g., HER2BATs consistently produce greater amounts of Th1 cytokines against low HER2-expressing MDAMB231 cells than high HER2-expressing SKBR3 cells even though cytotoxicity levels are similar (data not shown). And similar to BATs armed with anti-CD20 × anti CD3 BiAb (13), CS1 BATs remained active even in the presence of high concentrations of added elotuzumab. The ability of BiAbs to activate armed T cells in response to very low levels of receptor expression and/or differences between the affinity of soluble elotuzumab vs. its affinity as part of the BiAb bound to T cell receptors on the ATC are the likely reasons for maintaining killing in the presence of the targeting monoclonal antibody.

Arming of ATC with 25–50 ng of CS1Bi/10^6^ ATC was shown to kill MM targets at E:T as low as 1:1. Our prior studies show that ATC exhibit high levels of BiAb-mediated specific cytotoxicity as early as 6 days and as long as 18 days of culture. We have shown that ATC from patients can be armed with anti-CD3 × anti-HER2 BiAb to treat metastatic breast (5) and metastatic prostate (6) cancers, anti-CD3 × anti-CD20 BiAb to treat non-Hodgkin's lymphoma (7, 14) and MM (10), and anti-CD3 × anti-GD2 BiAb to treat neuroblastoma and osteosarcoma (15). All of the BiAb ATC combinations consistently enhance specific cytotoxicity above that seen in unarmed ATC (9, 13, 16, 17). Furthermore we showed that purified CD8 and CD4 populations could be armed with BiAb and mediate specific cytotoxicity, although we did not test T cell populations for antigen-specific cytotoxicity (9). It is clear that BATs are serial killers (8) and persist in the patients for weeks after infusions (5); in the former study, we tested for persistence of the HER2Bi on the surface of the T cells in serial killing assays and showed that the BiAb not only persists on the cell surface, but that a decreasing amount of BiAb on the surface passed on to the dividing daughter cells allows them to use the BiAb to kill again (8). Furthermore, arming with low doses of BiAb enables not only multiple serial killings but also continuous release of IFN-γ, TNF-α, and GM-CSF. The clinical immune evaluation studies in metastatic breast cancer, metastatic prostate cancer, non-Hodgkin's lymphoma and MM showed the induction of Th_1_ cytokine patterns with elevated levels of IFN-γ, TNF-α, GM-CSF, and IL-12 in the patients with increases in IP-10, and decreases in IL-8 (5, 6, 10, 18). A more recent study on immune transfer after stem cell transplant showed evidence for not only transfer of established anti-breast cancer immunity but the development of cellular and humoral immunity to other epitopes and well as other tumor antigens (19).

Other approaches to redirect T cell activity to treat MM include bispecific antibodies, non-gene and gene-modified T cell therapies (20–22), and chimeric antigen receptor (CAR)-expressing NK cells (23), from which several clinical trials have shown promising results in terms of response rate and/or duration. As shown with BLINCYTO^®^ and CAR-T products in other hematologic malignancies (24), the major side effects in MM pts have been CRS and neurotoxicity (20, 21) that occur due to the systemic nature of the target cell and difficulties in controlling both the dose and activity of the therapies. Therefore, the CS1-BATs approach is a highly promising alternative for use against MM due to the lack of toxicity demonstrated by previous BATs studies in solid tumors, NHL and MM, combined with the ability to more precisely control potency via adjusting (i) the amount BiAb used to arm the ATC, (ii) the cell dose per infusion, and (iii) the number and frequency of infusions. A clinical trial for refractory MM would be unique in that billions of CS1-BATs could be infused multiple times with minimal toxicities with or without a stem cell transplant in patients with resistant disease with the goal of reducing the tumor burden to attain a MRD status. Such a long-term strategy would lead to not only improved quality life for patients suffering from refractory MM but would lead to potential cures by immunologically eliminating the “last malignant plasma cell” using the endogenous immune system.

## Data Availability Statement

The raw data supporting the conclusions of this article will be made available by the authors, without undue reservation, to any qualified researcher.

## Ethics Statement

This study was approved by the University of Virginia (UVA) Institutional Review Board (IRB)#18904. All subjects gave written informed consent in accordance with the Declaration of Helsinki.

## Author Contributions

LL and AT conceived the use of CS1-BATs for treating MM. MH designed, validated, and implemented the flow cytometry-based cytotoxicity assay. AE, LA, and ED contributed to sample preparation and execution of experimental procedures. MH provided methods, data analysis, and figures. LL, MH, and AT authored the manuscript.

## Conflict of Interest

LL and MH are co-founders of TransTarget, Inc. AT is co-founder of Novo Immune Platform. The remaining authors declare that the research was conducted in the absence of any commercial or financial relationships that could be construed as a potential conflict of interest.
